# A Novel Object Detection Algorithm Combined YOLOv11 with Dual-Encoder Feature Aggregation

**DOI:** 10.3390/s25237270

**Published:** 2025-11-28

**Authors:** Haisong Chen, Pengfei Yuan, Wenbai Liu, Fuling Li, Aili Wang

**Affiliations:** 1 School of Integrated Circuit, Shenzhen Polytechnic University, Shenzhen 518115, China; hschen@szpu.edu.cn; 2 Heilongjiang Province Key Laboratory of Laser Spectroscopy Technology and Application, Harbin University of Science and Technology, Harbin 150080, China; 2510603099@stu.hrbust.edu.cn (P.Y.); 2320610204@stu.hrbust.edu.cn (W.L.); 2420610100@stu.hrbust.edu.cn (F.L.)

**Keywords:** object detection, YOLOv11, dual-encoder cross-attention, dual-encoder feature aggregation

## Abstract

To address the limitations of unimodal visual detection in complex scenarios involving low illumination, occlusion, and texture-sparse environments, this paper proposes an improved YOLOv11-based dual-branch RGB-D fusion framework. The symmetric architecture processes RGB images and depth maps in parallel, integrating a Dual-Encoder Cross-Attention (DECA) module for cross-modal feature weighting and a Dual-Encoder Feature Aggregation (DEPA) module for hierarchical fusion—where the RGB branch captures texture semantics while the depth branch extracts geometric priors. To comprehensively validate the effectiveness and generalization capability of the proposed framework, we designed a multi-stage evaluation strategy leveraging complementary benchmark datasets. On the M^3^FD dataset, the model was evaluated under both RGB-depth and RGB-infrared configurations to verify core fusion performance and extensibility to diverse modalities. Additionally, the VOC2007 dataset was augmented with pseudo-depth maps generated by Depth Anything, assessing adaptability under monocular input constraints. Experimental results demonstrate that our method achieves mAP50 scores of 82.59% on VOC2007 and 81.14% on M3FD in RGB-infrared mode, outperforming the baseline YOLOv11 by 5.06% and 9.15%, respectively. Notably, in the RGB-depth configuration on M^3^FD, the model attains a mAP50 of 77.37% with precision of 88.91%, highlighting its robustness in geometric-aware detection tasks. Ablation studies confirm the critical roles of the Dynamic Branch Enhancement (DBE) module in adaptive feature calibration and the Dual-Encoder Attention (DEA) mechanism in multi-scale fusion, significantly enhancing detection stability under challenging conditions. With only 2.47M parameters, the framework provides an efficient and scalable solution for high-precision spatial perception in autonomous driving and robotics applications.

## 1. Introduction

Binocular vision ranging technology [1], as a classical passive visual perception method, provides fundamental spatial position references for target detection through image disparity analysis [2], establishing the foundation for visual perception in autonomous driving [3], robotic navigation [4], and other fields. However, in complex scenarios involving low illumination, occlusion, and texture-sparse environments, unimodal visual detection faces severe challenges: image brightness attenuation, feature blurring, and high false detection rates significantly limit detection accuracy [5]. Consequently, multi-sensor fusion technology has emerged as a core pathway to enhance target detection robustness and precision by integrating complementary information from LiDAR, infrared, RGB, and other sensors.

As a key direction for improving detection accuracy, multi-sensor fusion technology has been extensively explored in recent research. Zhou et al. [6] conducted a comprehensive survey on RGB-D salient object detection (SOD), systematically categorizing existing methods into traditional and deep learning-based approaches and highlighting benchmark datasets such as NLPR and SIP. Their work emphasizes the complementary nature of RGB and depth modalities but notes persistent challenges in handling low-quality depth data and the need for effective fusion strategies. This survey underscores the importance of robust multimodal integration for real-world applications, though it primarily reviews existing works without proposing novel fusion mechanisms, limiting its direct applicability to dynamic environments. Peng et al. [7] explored early fusion strategies in RGB-D SOD by simply concatenating RGB and depth data at the input or feature level. This approach is straightforward but often fails to exploit modal differences, leading to performance degradation in complex scenes due to its inability to adapt to varying sensor qualities. While early fusion reduces computational overhead by processing modalities jointly, it lacks the flexibility to handle inconsistencies between RGB and depth data, making it vulnerable to noise and occlusion commonly encountered in practical scenarios. Han et al. [8] investigated late fusion methods, which process RGB and depth modalities independently before combining predictions. This strategy preserves modality-specific features but may miss cross-modal interactions during early processing stages, resulting in suboptimal fusion for objects with similar appearances. Late fusion enhances robustness by leveraging separate feature extractors, yet it often requires careful calibration to align modal outputs and can struggle with real-time applications due to its sequential processing pipeline.

To overcome the limitations of the aforementioned early and late fusion strategies, researchers have turned to more flexible intermediate fusion strategies. Li et al. [9] proposed ASIF-Net, an interweave fusion scheme that incorporates an attention mechanism to dynamically steer the complementarity between RGB and depth features. The model employs a dual-branch architecture with cross-modal attention, enhancing feature representation by selectively emphasizing informative regions. ASIF-Net improves accuracy in occluded and low-light conditions by integrating attention-weighted features, but its complex modules increase computational costs, and it may still face challenges with deeply occluded objects. Chen and Li [10] developed the PCF network, which uses a complementarity-aware fusion module to integrate cross-modal features through cross-level interactions. This approach reduces fusion ambiguity by explicitly modeling modal dependencies. PCF achieves higher accuracy by capturing both low-level details and high-level semantics, but it requires meticulous parameter tuning and can be sensitive to depth map quality, limiting its generalization in diverse environments. Chen et al. [11] introduced the MMCI network, incorporating multi-scale multi-path fusion to handle cross-modal interactions. This method enhances feature richness by combining multiple pathways but may incur increased memory usage. MMCI effectively addresses fusion ambiguity by leveraging hierarchical features, yet its resource-intensive design hinders deployment on resource-constrained devices, and it assumes uniform modal reliability, making it prone to errors with noisy inputs. Liu et al. [12] proposed the Visual Saliency Transformer (VST), which applies self-attention mechanisms to capture long-range dependencies for SOD. This model excels in handling large-scale scenes by modeling global context. VST improves performance in complex backgrounds through transformer-based encoding, but its focus on high-level semantics may overlook fine-grained edge details, and it demands significant computational resources, reducing practicality for real-time applications. Chen et al. [13] designed EM-Trans, a novel edge-aware framework that decomposes features into high-frequency (edge) and low-frequency (body) components within a dual-band framework. Its Cross-Attention Complementarity Exploration (CACE) module enriches features by exploiting multimodal complementarity. EM-Trans significantly enhances boundary accuracy and robustness in challenging conditions like occlusion and low light through explicit edge modeling, but the dual-band decomposition and attention mechanisms increase computational overhead.

The above research focuses on feature-level enhancement and decomposition strategies, aiming to achieve more refined and efficient fusion through signal preprocessing, feature separation, and lightweight operators. However, these methods often rely on manually designed fusion rules and lack dynamic environmental perception capabilities.

For refined design of infrared-visible light fusion, Pan et al. [14] first introduced diffusion models into fusion tasks, proposing the dual-branch DANet network, significantly improving the support capability of fused images for detection tasks. Li et al. [15] proposed the heterogeneous dual-branch multi-cascade network DCTNet: the CNN branch extracts visible light local textures through Residual Dense Blocks (RDB), while the Transformer branch captures infrared global thermal distribution through Residual Transformer Blocks (RTB), with the Adaptive Fusion Interaction Module (AFIM) combining Multi-Scale Edge Attention (MSEA) and Spatial-Channel Attention (SCA) for dynamic feature fusion. Hu et al. [16] proposed the PFCFuse network, where the Poolformer branch extracts global features through pooling attention, the CNN branch extracts local details through depthwise separable convolution, and cross-modal attention weighted fusion preserves infrared thermal targets and visible light textures. Huan et al. [17] proposed the target-aware Taylor expansion approximation network T2EA, which approximates target local features through Taylor expansion to enhance infrared small target representation, achieving a 98.7% detection rate and 15.6% reduction in false alarm rate on infrared small target datasets. Research teams [18] proposed the HitFusion network utilizing Transformers to capture high-order feature interactions: cross-modal attention dynamically models infrared-visible light feature correlations, while multi-scale Transformer blocks fuse features at different levels to enhance semantic consistency. Research teams [19] designed a dual-branch autoencoder + reversible high-frequency encoding network, using reversible neural networks to encode high-frequency features and avoid information loss, with high-frequency features enhanced through element-wise multiplication and low-frequency features preserved through channel concatenation. Research teams [20] proposed a prior semantic-guided fusion method combining semantic segmentation tasks to guide fusion: parallel semantic branches perceive modality-specific semantics through Residual Feature adaptive Modulation (RFaM), while Multi-level Representation adaptive Fusion (MRaF) modules fuse low-frequency prior semantics with high-frequency details.

While these advanced fusion strategies significantly improve feature representation, they often introduce high computational complexity or require auxiliary tasks. This creates a tension between fusion performance and practical deployability, particularly for real-time applications.

Recently, the combination of YOLO [21] series algorithms with multi-sensor fusion [22] has further promoted high-precision detection system applications: Zeng et al. [23] combined multi-sensor fusion with an improved YOLOv5 algorithm, incorporating lightweight modifications and attention mechanisms, replacing traditional manual feature extraction and significantly enhancing obstacle detection capability in multi-target and occlusion scenarios.

Table 1 lists some representative studies, summarizes the methods they adopted and their main advantages, to highlight the characteristics and limitations of various fusion strategies.

Despite significant progress, current multimodal detection systems face three persistent challenges: (1) Limited dynamic adaptability in cross-modal feature interaction under varying environmental conditions [24]; (2) High computational overhead from complex fusion operators conflicting with real-time requirements; (3) Architectural incompatibility when extending lightweight detectors to multisensory input without redesigning the fusion mechanism [25].

These studies fully validate that multi-sensor fusion can effectively compensate for unimodal detection deficiencies, providing feasible technical pathways for target detection accuracy improvement in complex environments.

1. We propose an improved dual-branch RGB-D fusion object detection framework based on YOLOv11. By designing a symmetric network structure, RGB images and depth maps are processed in parallel. This enables the model to simultaneously leverage the rich texture and semantic information from RGB data and the geometric structural information provided by depth data, significantly enhancing detection performance in complex environments such as low illumination, occlusion, and texture-sparse scenarios.
2. We design a Dynamic Branch Enhancement (DBE) module that incorporates a combined spatial-channel attention mechanism. This module achieves adaptive weighting of cross-modal features, dynamically enhancing complementary information while suppressing redundant or noisy feature activations, thereby improving the model’s robustness and feature representation capability.
3. We introduce a Dual-Encoder Attention (DEA) module, which consists of two sub-modules: the Dual-Encoder Cross-Attention (DECA) for refining features within each modality, and the Dual-Encoder Feature Aggregation (DEPA) for effectively integrating cross-modal information. This hierarchical design enables multi-scale feature fusion, strengthening the model’s ability to perceive and represent multi-modal information.
4. We employ the Depth Anything model to generate pseudo-depth maps from single RGB images, constructing a pseudo-RGBD training set. This approach effectively addresses the limitation of lacking real depth sensor data in conventional visible-light datasets, providing a feasible solution for training and evaluating RGB-D fusion detection models under monocular data constraints.

## 2. Methods

**Figure 1:**
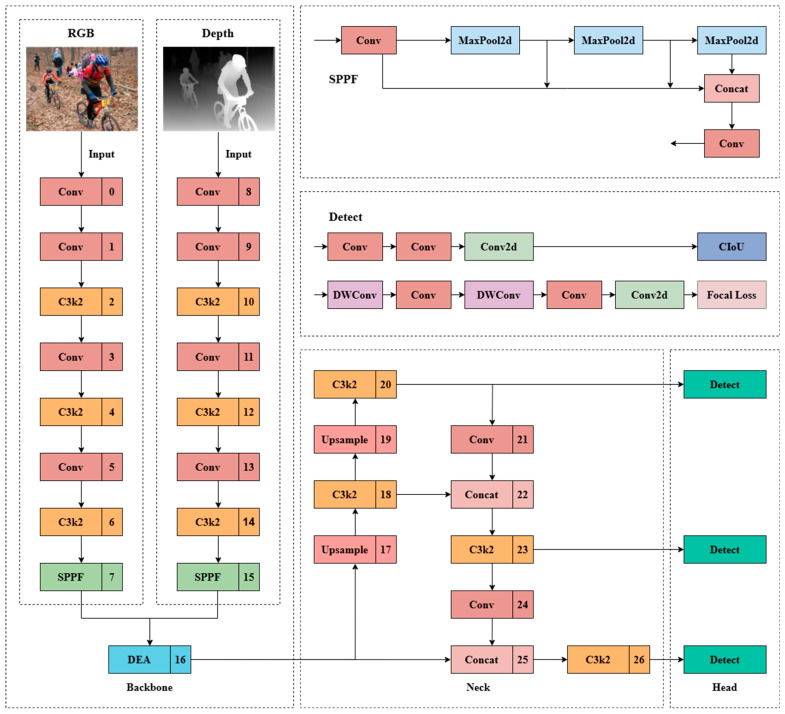
The structure of the improved YOLOv11-based dual-branch RGB-D fusion.

Recognizing the inherent limitation of conventional YOLO architectures in processing multi-modal data, we propose a novel dual-branch framework founded on YOLOv11.This design is engineered to synergistically process both RGB images, which provide rich texture and semantic details, and pixel-aligned depth maps, which convey indispensable geometric and structural information. The parallel processing of these complementary modalities enables our model to achieve robust pedestrian detection and accurate binocular distance estimation, even in challenging scenarios characterized by occlusion, scale variation, and illumination changes. The overall architecture, conceptually depicted in Figure 1, establishes two independent yet symmetric pathways before integrating their feature representations for subsequent prediction.

RGB Branch: This branch incorporates the CSPDarknet53 backbone from YOLOv11, selected for its effective balance between computational efficiency and representational capacity. It extracts hierarchical spatial-semantic features: the initial layers capture low-level texture and edge information, while deeper layers encode high-level semantic context essential for distinguishing pedestrians from cluttered backgrounds and supporting accurate object classification.

Depth Branch Operating in parallel, this branch processes the corresponding depth input using an identical CSPDarknet53 structure. This symmetry ensures that features from both modalities are extracted at consistent semantic levels, thereby facilitating effective fusion. The depth branch specializes in capturing geometric priors and spatial relationships, providing direct cues about object proximity (e.g., brighter intensity indicates closer distance) and precise occlusion boundaries—often ambiguous in RGB alone. This capability is particularly valuable for resolving overlapping pedestrians and improving the localization of distant or small objects.

Through this carefully designed dual-branch architecture, our model learns rich interactive representations that leverage the distinct strengths of each modality throughout the hierarchical feature extraction process, rather than simply concatenating features.

### 2.1. Dual-Encoder Attention

**Figure 2:**
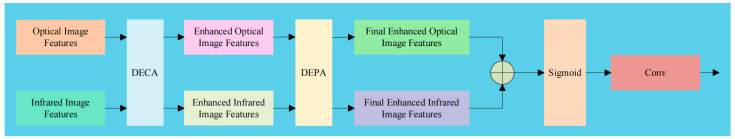
The structure of DEA module.

To achieve effective interaction and fusion between the RGB and depth modalities [26], we design a dedicated Dual-Encoder Attention (DEA) module. As illustrated in Figure 2, the DEA module consists of two core sub-modules: the Dual-Encoder Cross-Attention (DECA) and the Dual-Encoder Feature Aggregation (DEPA). This hierarchical design enables the model to first refine features within each modality and then perform cross-modal feature fusion, effectively capturing both intra-modal and inter-modal dependencies [27].

#### 2.1.1. Dual-Encoder Cross-Attention

**Figure 3:**
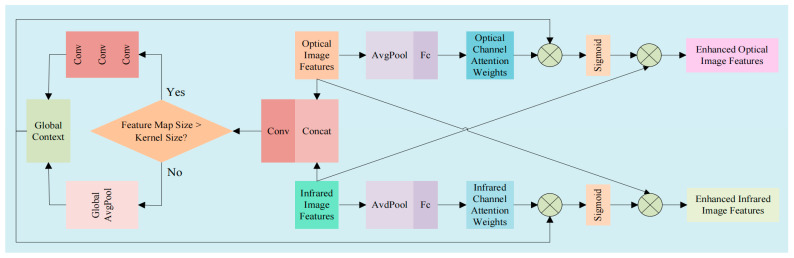
The structure of DECA module.

The DECA sub-module is responsible for enhancing the features within each modality by leveraging channel-wise attention mechanisms. Specifically, it takes the intermediate features from both the RGB and depth branches as inputs. For each modality, it first applies a convolutional layer to enhance feature representation, followed by a channel attention block that consists of global average pooling, fully connected layers, and a sigmoid activation function. This process generates modality-specific attention weights that emphasize informative channels and suppress less useful ones. The enhanced features are obtained by multiplying the original features with the computed attention weights. The outputs of the DECA sub-module are thus refined features for both modalities, which are then fed into the subsequent DEPA sub-module for cross-modal interaction. The structure of DECA as shown in Figure 3.

#### 2.1.2. Dual-Encoder Feature Aggregation

**Figure 4:**
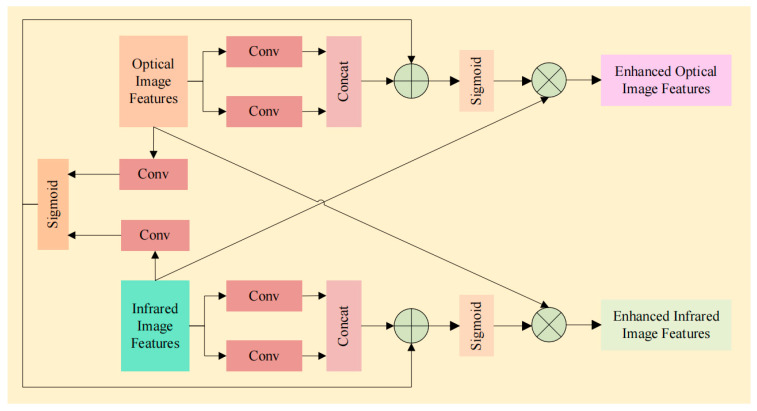
The structure of DEPA module.

The DEPA sub-module performs the actual fusion of the refined RGB and depth features. It first applies convolutional layers to further process the features from each modality. The processed features are then concatenated along the channel dimension, followed by a series of operations including convolutional layers, sigmoid activation, and element-wise multiplication. This allows the model to learn a shared representation that effectively combines complementary information from both modalities. The output of the DEPA sub-module is a fused feature map that incorporates both texture details from the RGB modality and geometric information from the depth modality. The structure of DEPA as shown in Figure 4.

The proposed DEA module is inserted at multiple levels of the dual-stream architecture, enabling multi-scale feature fusion. This design ensures that the model can leverage the strengths of both modalities at different semantic levels, leading to more robust and accurate pedestrian detection and distance estimation. The overall operation of the DEA module is summarized by Equation (1):

(1) $$F_{\mathrm{fusion}}=\mathrm{DEPA}\left(\mathrm{DECA}\left(F_{\mathrm{RGB}}\right),\mathrm{DECA}\left(F_{D}\right)\right)$$

where $F_{\mathrm{RGB}}$ and $F_{D}$ are the input features from the RGB and depth branches, respectively, and $F_{\mathrm{fusion}}$ is the output fused feature.

### 2.2. Loss Function

In pedestrian detection tasks using the VOC2007 dataset, a significant challenge arises from the inherent class imbalance and the presence of hard samples. Certain pedestrian instances may be difficult to detect accurately due to factors such as occlusion, poor lighting conditions, or viewpoint changes, causing the model to overlook these challenging examples during training and consequently underperform on such cases. The standard Cross Entropy Loss, which is a common choice for classification tasks, is formally described by Equation (2):

(2) $$L_{ce}\left(y,\hat{p}\right)=\left\{\begin{array}{cc} -\log\left(\hat{p}\right), & \mathrm{if}\;y=1\\ -\log\left(1-\hat{p}\right), & \mathrm{if}\;y=0 \end{array}\right.$$

Therefore, the two expressions above can be combined and written in the unified form of Equation (3):

(3) $$L_{ce}=-\log\left(p_{t}\right)$$

where $p_{t}$ denotes the model’s estimated probability for the ground-truth class. A larger $p_{t}$ indicates a more confident and accurate classification.

However, the cross-entropy loss treats all samples equally. When a substantial imbalance exists between positive and negative samples (e.g., a vast number of background negatives versus fewer pedestrian positives), the loss function becomes dominated by the majority class. This leads the model to develop a bias towards the prevalent class, thereby degrading detection performance for the minority class, i.e., pedestrians.

To mitigate this issue, an intuitive improvement is the balanced cross-entropy loss. It incorporates a weighting factor to adjust the contribution of each class to the total loss, and is formulated as shown in Equation (4):

(4) $$L_{balanced}=\frac{1}{N}\left(\sum_{y_{i}=1}^{m}-\alpha \log\left({\hat{p}}_{i}\right)+\sum_{y_{i}=0}^{n}-\left(1-\alpha \right)\log\left(1-{\hat{p}}_{i}\right)\right)$$

where the weight α is typically set such that $\frac{\alpha }{1-\alpha }=\frac{n}{m}$, countering the imbalanced distribution of samples.

Building upon this, Focal Loss is introduced to further enhance the model’s focus on hard, misclassified examples. It dynamically scales the standard cross entropy loss as formally defined in Equation (5), reducing the contribution of easy-to-classify samples and increasing the focus on hard negatives:

(5) $$L_{fl}=-\alpha_{t}{\left(1-p_{t}\right)}^{\gamma }\log\left(p_{t}\right)$$

In this formulation: $\alpha_{t}$ is a class-balancing factor that addresses the fundamental imbalance between positive and negative samples by assigning a higher weight to the minority class (e.g., pedestrians); ${\left(1-p_{t}\right)}^{\gamma }$ is the modulating factor designed to down-weight the loss contribution from well-classified, easy examples (where $p_{t}$ is large) and focus training on hard, misclassified samples (where $p_{t}$ is small); and γ is a tunable focusing parameter that controls the rate at which easy examples are de-emphasized.

The value $p_{t}$ also reflects the difficulty of a sample: a larger $p_{t}$ corresponds to an easy sample, while a smaller $p_{t}$ indicates a hard sample. Focal Loss effectively increases the relative contribution of hard samples to the total loss, directing the model’s learning focus towards these challenging instances and thereby improving overall classification and detection performance, particularly for occluded, small-scale, or poorly illuminated pedestrians.

The efficacy of Focal Loss stems not only from its re-weighting scheme but also from its inherent dynamic gradient adjustment mechanism. Specifically, the update of model parameters is governed by the gradient of the loss function with respect to the parameters, as given in Equation (6):

(6) $$\omega =\omega -\eta \frac{\partial L}{\partial \omega }$$

For Focal Loss, the gradient is smaller when $p_{t}$ is large (easy samples) and more substantial when $p_{t}$ is small (hard samples). This ensures the optimization process concentrates on hard examples, ultimately enhancing the model’s robustness and accuracy in complex real-world scenarios and leading to superior performance in downstream tasks such as stereo vision-based distance estimation.

## 3. Results

To comprehensively validate the effectiveness of the dual-branch RGB-D fusion framework, this study designs a stepwise experimental process based on the multimodal characteristics of the M^3^FD dataset (which includes RGB, depth, and infrared data). Firstly, we utilize the RGB and depth modalities of M^3^FD for training to test the model’s basic detection accuracy in a real multimodal environment. Secondly, to address the challenge of missing depth data in practical applications, the VOC2007 dataset is introduced, and pseudo-depth maps are generated via Depth Anything to construct an RGB-pseudo-depth training set, thereby evaluating the model’s generalization capability. Furthermore, we extend the evaluation to the combination of RGB and infrared modalities within the M^3^FD dataset, validating the framework’s fusion extensibility for non-depth modalities (such as infrared information). This multimodal, multi-scenario experimental design ensures the comprehensiveness and logical rigor of the evaluation.

### 3.1. Experimental Configuration and Model Evaluation Metrics

The computer configuration used for model training was an Intel Core i5-13600KF CPU (14 cores, 20 threads, and the Intel Core i5-13600KF CPU was sourced from the open retail market. The manufacturer is Intel Corporation, headquartered in Santa Clara, CA, USA), 64 GB of RAM, and an NVIDIA GeForce RTX 4070 graphics card (12 GB of GDDR6X video memory). The system operated on Windows10, with CUDA version 11.8 and cuDNN version 8.6.0. The training parameters were set as follows: image size = 640, cache = True, epochs = 200, batch = 32, device = 0, workers = 4, patience = 50.

### 3.2. Evaluation Indicators

To quantitatively evaluate the performance of the proposed multi-modal object detection framework, this study employs a comprehensive set of widely adopted metrics in object detection research. These metrics provide rigorous mathematical formulations to assess different aspects of detection accuracy and robustness.

Precision measures the proportion of correctly detected positive instances among all detected objects, as defined in Equation (7):

(7) $$\mathrm{Precision}=\frac{TP}{TP+FP}$$

where TP denotes True Positives and FP represents False Positives.

Recall quantifies the ability of the model to identify all relevant ground-truth objects, as given in Equation (8):

(8) $$\mathrm{Recall}=\frac{TP}{TP+FN}$$

where FN denotes False Negatives.

Mean Average Precision serves as the primary evaluation criterion, providing a comprehensive measure of detection accuracy across all categories. The mAP is calculated by averaging the Average Precision (AP) values over all object categories, as formulated in Equation (9):

(9) $$\mathrm{mAP}=\frac{1}{n}\sum_{k=1}^{k=n}AP_{k}$$

where N denotes the number of object categories and $AP_{k}$ represents the Average Precision for the k-th category.

These metrics collectively provide a multi-faceted evaluation framework that assesses both classification accuracy (through precision and recall) and localization precision (through mAP at various IoU thresholds). The mathematical rigor of these formulations ensures objective comparison with state-of-the-art methods, while their widespread adoption in the research community enables meaningful benchmarking of the proposed method’s performance.

### 3.3. Introduction to the Dataset

To comprehensively validate the effectiveness and generalization capability of the proposed multimodal fusion framework, our evaluation leverages two complementary benchmark datasets, each serving a distinct purpose. The M^3^FD dataset provides aligned RGB, depth, and infrared modalities. This experiment primarily utilizes its RGB-depth combination to validate the core fusion mechanism, while additionally employing the RGB-infrared combination to test the framework’s extensibility to other modalities. As a supplement, the VOC2007 dataset, which contains only RGB modality, is transformed into a pseudo-RGBD dataset via Depth Anything. This demonstrates the framework’s adaptability and effectiveness under the practical constraint of monocular image input.

#### 3.3.1. M^3^FD Dataset

**Figure 5:**
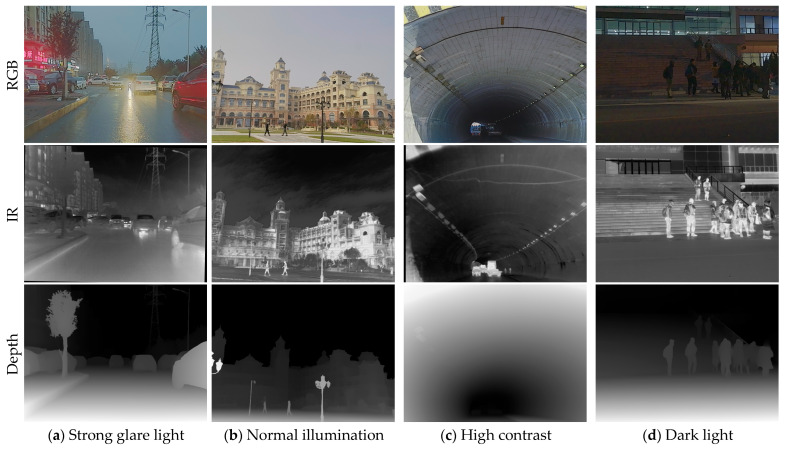
M^3^FD dataset and examples of transformed depth images. The first row in the figure is an RGB image, the second row is an infrared image, and the third row is a depth image.

The M^3^FD (Multi-scenario Multi-Modality Benchmark) dataset is a large-scale, meticulously annotated benchmark dataset specifically designed for infrared and visible image fusion and its downstream tasks, such as object detection. Developed by Dalian University of Technology, this dataset contains a total of 4200 precisely registered infrared and visible image pairs (totaling 8400 images), all with a resolution of 1024 × 768 pixels. Sample images from the dataset are shown in Figure 5.

**Figure 6:**
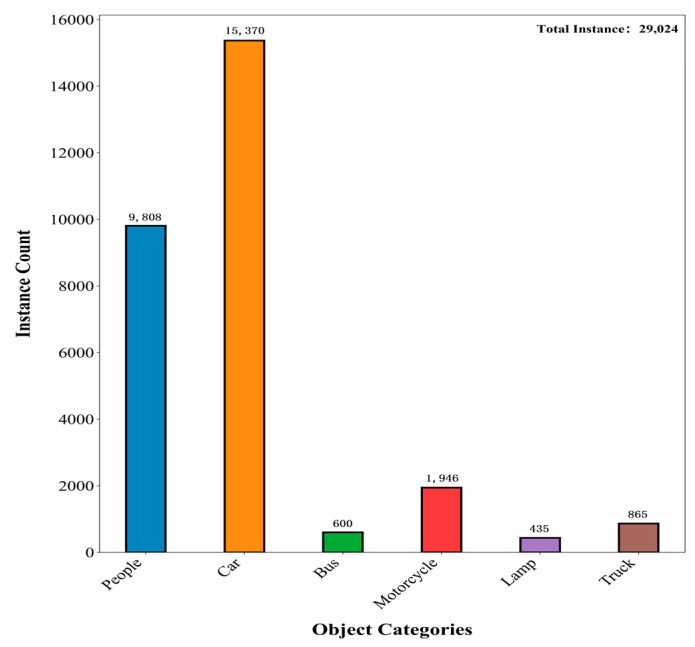
The quantity distribution of each category in the M^3^FD datasets.

To ensure high data quality, the acquisition system was rigorously calibrated, and pixel-level cross-modal alignment was achieved through manual correction. The dataset encompasses diverse real-world scenarios, including campuses, urban roads, and tourist areas, and specifically incorporates challenging conditions such as low illumination, strong glare, and dynamic objects, significantly enhancing its complexity and practical utility. The dataset includes six object categories: People, Car, Bus, Motorcycle, Lamp, and Truck. The category distribution of the dataset is illustrated in Figure 6.

#### 3.3.2. VOC2007 Dataset

PASCAL VOC 2007 (Visual Object Classes 2007), released as part of the PASCAL Visual Object Classes challenge in 2007, has been extensively utilized for evaluating the performance of image classification, object detection, and semantic segmentation algorithms. The dataset consists of 9963 natural images annotated with 20 common object categories, which are broadly divided into four major groups: vehicles, animals, indoor objects, and persons.

**Figure 7:**
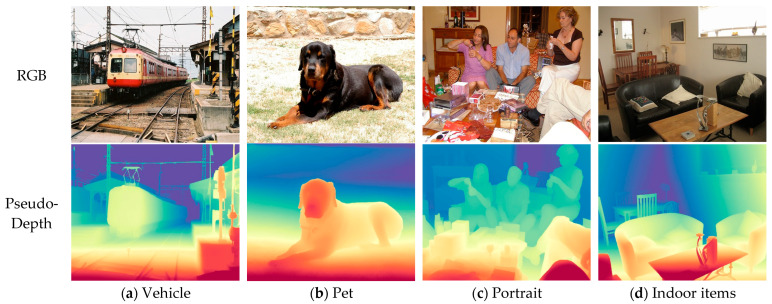
VOC2007 dataset and examples of transformed depth images.

In order to adapt the algorithm—which was originally designed for dual-sensor input—to the available optical-image-only dataset, this study employs the Depth Anything model to generate corresponding depth maps from single RGB images, thereby constructing a pseudo dual-modal training set. The Depth Anything model, a robust monocular depth estimation method pre-trained on large-scale datasets, is applied to each input image to infer per-pixel depth information without requiring additional camera parameters. The resulting depth maps are aligned spatially with the original images at the same resolution and viewing angle, preserving geometric consistency. Both the original RGB images and the synthesized depth maps are then normalized to a [0, 1] range and integrated, either by channel-wise concatenation to form a four-channel RGB-D representation or as parallel inputs into the dual-branch fusion network. This augmentation effectively simulates a dual-sensor data environment, enabling the effective training and evaluation of the multimodal fusion framework under the constraint of using a conventional visible-light image dataset. Sample images from the dataset are illustrated in Figure 7.

**Figure 8:**
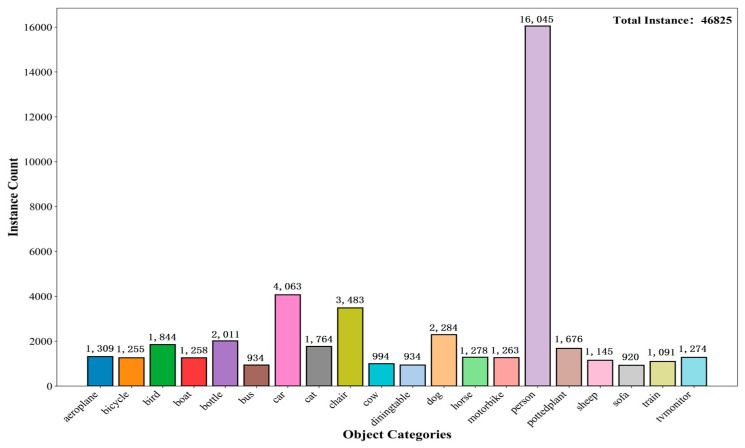
The quantity distribution of each category in the VOC2007 datasets.

Each image is accompanied by high-quality XML-format annotation files that provide detailed information, including object categories, bounding box coordinates, truncation status, and difficulty level for recognition. The dataset is officially pre-divided into training, validation, and test sets, ensuring fair and reproducible comparisons across different studies. The primary evaluation metric is the mean Average Precision which has become the de facto standard for measuring the performance of object detection models. The category distribution of the dataset is illustrated in Figure 8.

### 3.4. Comparison Experiment

After completing the model improvements, a comparative analysis was carried out between the proposed model and several current mainstream object detection models. Comparative experiments are conducted across three dimensions: testing RGB-depth fusion on M^3^FD to validate fundamental multimodal performance, evaluating RGB-pseudo-depth fusion on VOC2007 to assess generalization capability, and further examining RGB-infrared fusion on M^3^FD to verify modality extensibility. The evaluation comprehensively considered multiple performance aspects, including precision, recall, mAP50 and mAP50-95 computational complexity, and number of parameters, to objectively quantify the performance gains achieved by the improved model.

The improved dual-branch fusion model based on YOLOv11 demonstrates strong competitiveness in infrared-visible cross-modal detection tasks on the challenging M^3^FD benchmark. On the M^3^FD dataset, we compared the YOLO series, RT-DETR, TarDAL [28], and MMDetection-DS algorithms. As shown in Table 2, the model achieves an mAP50 of 81.14% on the M^3^FD dataset. This represents a 9.15% improvement over the original YOLOv11 model, while performing comparably to the computationally intensive RT-DETR model (81.20%). It also significantly outperforms other YOLO series models: YOLOv5n achieves 73.12%, YOLOv8n reaches 74.91%, YOLOv10n attains 76.04%, YOLOv12n scores 70.88%, and YOLOv13n obtains 61.94%. This performance breakthrough stems from the dual-branch architecture specifically designed for multimodal scenarios—the visible branch precisely extracts texture details, while the complementary infrared branch effectively analyzes thermal signatures. The synergistic operation of the DEA modules enhances cross-modal feature complementarity in challenging environments such as strong glare and low-light conditions, effectively addressing the performance degradation issues encountered by traditional unimodal models in extreme illumination scenarios.

**Figure 9:**
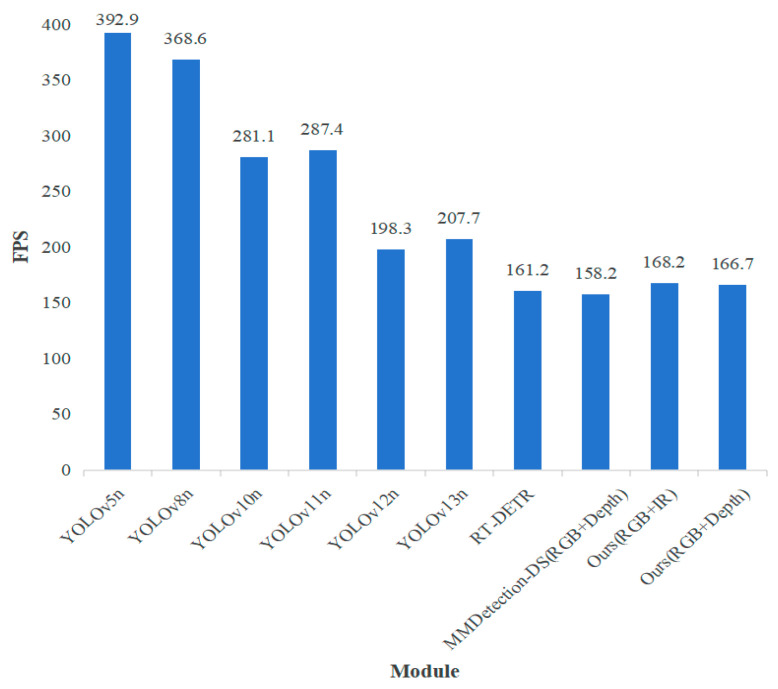
FPS comparison of different models.

When evaluating the practicality and deployment potential of object detection models, inference speed is a key metric equally as important as detection accuracy. A high frame rate (FPS) is a prerequisite to ensure the system can respond promptly to environmental changes, especially in real-time application scenarios such as autonomous driving and video surveillance. Therefore, comparing the FPS performance of different models can comprehensively reflect their efficiency advantages in real-world applications. As shown in Figure 9, our method achieves a good balance between detection accuracy and computational efficiency. Specifically, regarding the FPS comparison data: lightweight models (e.g., YOLOv5n and YOLOv8n), by virtue of their extremely simplified architectures, both exceed 300 FPS, demonstrating the inherent speed advantage of single-modal models. However, for complex tasks that require the fusion of multi-modal information (such as infrared or depth data) to enhance detection robustness, our method maintains excellent real-time performance while incorporating additional information. Our model based on RGB + IR achieves an FPS of 168.2, and the model based on RGB + Depth achieves an FPS of 166.7. This speed surpasses that of the multi-modal fusion solution MMDetection-DS (RGB + Depth, 158.2 FPS) and also outperforms the Transformer-based RT-DETR (161.2 FPS).

The improved dual-branch fusion model based on YOLOv11 proposed in this paper demonstrates significant advantages on the VOC2007 dataset. As shown in Table 3, the model achieves 82.59% on the key metric mAP50, surpassing not only the YOLOv11n model’s 77.53% but also outperforming other mainstream lightweight detectors: YOLOv5n at 56.40%, YOLOv8n at 77.21%, YOLOv10n at 62.46%, YOLOv12n at 77.17%, and YOLOv13n at 58.55%. It also substantially exceeds the RT-DETR model’s 75.88%. This improvement in accuracy stems from an innovative dual-modal processing architecture—where the model extracts texture and semantic features through a parallel RGB branch while the depth branch parses geometric structural information. Coupled with the core DEA modules, it effectively integrates the complementary advantages of both modalities.

In terms of model efficiency, despite the incorporation of depth computation branches and complex fusion mechanisms, the parameter count is controlled at 2.47M, remaining comparable to the original YOLOv11n’s 2.59M and significantly lower than RT-DETR’s 19.90M. The computational cost increased for cross-modal feature fusion, at 19.4 GFLOPs, is higher than other YOLO series models (6.3–8.1 GFLOPs) but remains substantially lower than RT-DETR’s 57.0 GFLOPs, reflecting a reasonable balance between accuracy improvement and computational burden. Overall, the experimental data validate the scientific soundness and practicality of the dual-branch RGB-D fusion architecture, providing an effective solution for robust visual perception in complex environments.

Fine-grained detection results on the M^3^FD dataset as shown in Table 4 demonstrate the model’s precise capture of thermal signature targets: people detection accuracy reaches 81.60%, representing a 15.55-percentage-point improvement over the original YOLOv11n, while bus detection accuracy increased by 3.17 percentage points. Particularly for infrared-sensitive objects, lamp detection accuracy reaches 74.76% (surpassing the YOLOv11n baseline by 16.38 percentage points), and truck detection accuracy improved by 5.76 percentage points. These advancements are directly attributable to the synergistic mechanism of the dual-branch architecture—the visible branch enhances texture details (e.g., vehicle contours), while the complementary modality branch strengthens thermal radiation feature perception (e.g., human heat sources). Coupled with the DEA module’s dynamic channel weighting strategy, the architecture maintains stable feature fusion capability even under intense glare interference.

The proposed dual-branch fusion model demonstrates comprehensive performance improvements in fine-grained object detection. As shown in Table 5 for the 20-category object detection task on the VOC2007 dataset, the model outperforms the original YOLOv11n in 18 categories, with particularly significant gains in traditionally challenging categories: bottle detection accuracy increased by 15.62 percentage points from 59.80% to 75.42%; chair detection improved by 10.19 percentage points from 59.97% to 70.16%; and pottedplant detection rose from 51.70% to 49.71%. Notably, the solution achieves substantial advances in geometrically structured objects where traditional unimodal models typically underperform: boat detection accuracy improved by 5.72 percentage points and diningtable detection increased by 10.27 percentage points. These results validate that the geometric prior information provided by the depth branch effectively compensates for the perceptual limitations of the RGB modality on texture-deficient targets.

**Figure 10:**
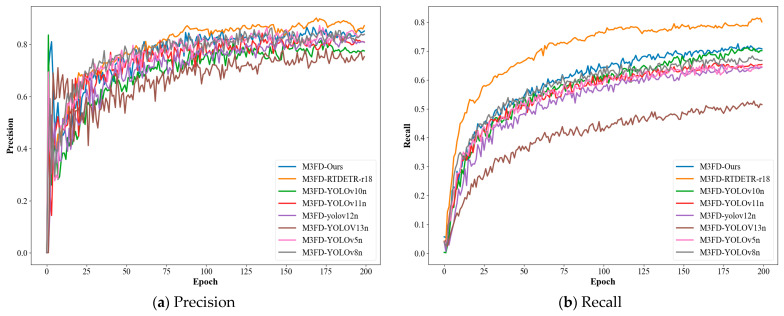

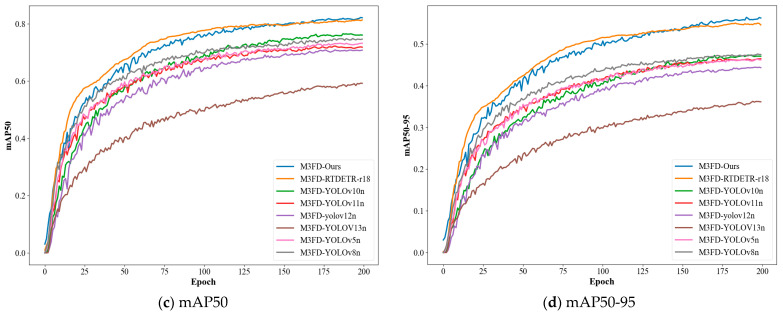
Performance Comparison of Object Detection Models on M^3^FD Dataset.

Figure 10 presents a comprehensive evaluation of the proposed model alongside several state-of-the-art detectors on the M^3^FD dataset, with performance metrics tracked across 200 training epochs. The results are organized in a 2 × 2 panel layout, comparing Precision, Recall, mAP50, and mAP50-95Precision, Recall, mAP@0.5, and mAP@0.5:0.95.

In the Precision curve (top-left), the proposed method (M^3^FD-Ours, blue line) demonstrates superior performance, achieving the highest convergence speed and stabilizing at approximately 0.92, significantly outperforming all other models. This indicates its exceptional ability to minimize false detections. The Recall curve (top-right) further shows that our method maintains a high and stable recall value, contrasting with the noticeable fluctuations observed in models such as M^3^FD-YOLOv5n.

For the primary detection metric mAP50 (bottom-left), the proposed model achieves the highest score of 81.14%, underscoring its strong generalization capability in complex scenarios involving cross-modal data. Similarly, in the more challenging mAP50-95 (bottom-right), which evaluates performance across multiple IoU thresholds, our method consistently retains leading performance, reflecting its robustness and accuracy under varying detection conditions.

Overall, the proposed model exhibits optimal performance across all evaluation metrics, with faster convergence and greater training stability compared to the other models. These results validate the effectiveness of the dynamic dual-branch fusion architecture and the use of pseudo-depth data augmentation in enhancing cross-modal object detection.

**Figure 11:**
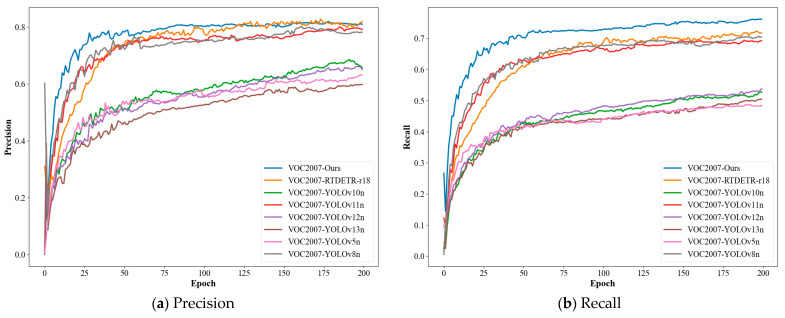

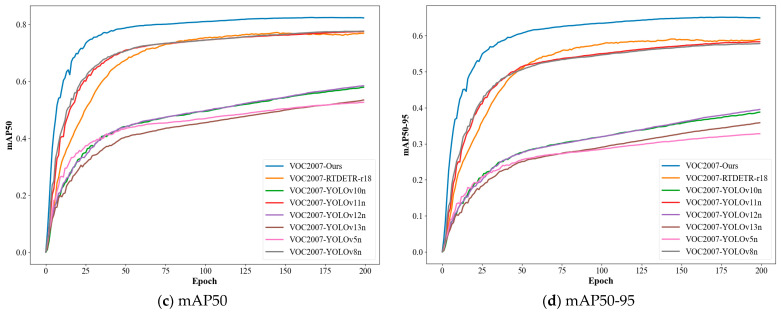
Performance Comparison of Object Detection Models on VOC2007 Dataset.

Figure 11 presents a comparative analysis of the training performance between the proposed model and several mainstream detection models on the VOC2007 dataset. The figure comprehensively evaluates all models across four key metrics—Precision, Recall, mAP50, and mAP50-95—over 200 training epochs.

In the Precision curve (top-left), the proposed method significantly outperforms all other compared models, demonstrating not only the fastest convergence speed but also the highest final stability (approximately 0.92), indicating the lowest false detection rate and optimal localization quality. The Recall curve (top-right) shows that the proposed method maintains a high recall level while achieving more stable convergence, markedly superior to the more fluctuating YOLOv5n series models. On the core detection metric mAP50, the proposed method leads with an outstanding score of 82.59%, validating the strong generalization capability of multimodal feature fusion in complex scenes. Moreover, in the more stringent comprehensive metric mAP50-95, the proposed method also maintains the highest performance, further demonstrating its robustness across different IoU thresholds.

Overall, the proposed model achieves optimal performance across all evaluation metrics, with faster convergence and stronger training stability, fully proving the effectiveness of the dynamic dual-branch fusion mechanism and the pseudo-depth data augmentation strategy.

### 3.5. Ablation Experiment

This study quantitatively validates the synergistic contributions of each innovative module in the dual-branch architecture through systematic ablation experiments. As shown in Table 6, when only the Focal Loss is incorporated into YOLOv11n (denoted as YOLOv11n + A), the model’s mAP50 on the VOC2007 dataset increases by 0.5 percentage points to 78.03%, indicating that this module effectively mitigates class imbalance issues. The independent adoption of the DEA interaction enhancement module (denoted as YOLOv11n + B) yields more substantial performance improvements: mAP50 significantly rises by 4.57 percentage points to 82.10%, while recall improves by 5.54 percentage points to 75.03%. This demonstrates the critical role of the dynamic cross-modal attention mechanism in enhancing adaptability to complex scenarios. When both modules are fully integrated (denoted as YOLOv11n + A + B), the model achieves optimal performance: mAP50 further increases to 82.59% and recall reaches 75.61%, representing a 5.06-percentage-point accuracy gain over the baseline model. These results confirm the positive synergistic effects between the modules.

To evaluate the contribution of different modal information and our fusion strategy, a comprehensive modality-wise ablation study was conducted, and the results are presented in Table 7. First, in the single-modality baseline tests, the visible modality achieved the best performance (71.89% mAP50, 50.37% mAP50-95), indicating its inherent advantage in daytime scenarios. The depth and infrared modalities achieved mAP50 scores of 67.23% and 68.70%, respectively, demonstrating their considerable feature representation capabilities as complementary information sources. More importantly, modality fusion led to a significant performance boost. When visible and depth information were fused, the mAP50 increased to 77.37%. Notably, the combination of visible and infrared modalities yielded the best result, achieving an mAP50 of 81.14%, which is an improvement of nearly 10 percentage points over using the visible modality alone. In terms of efficiency, all model variants maintained a similar parameter count, highlighting the compact design of our architecture. The increase in computational complexity (GFLOPs) for the dual-modality fusion is a reasonable trade-off between accuracy and efficiency.

### 3.6. Analysis of Test Results

**Figure 12:**
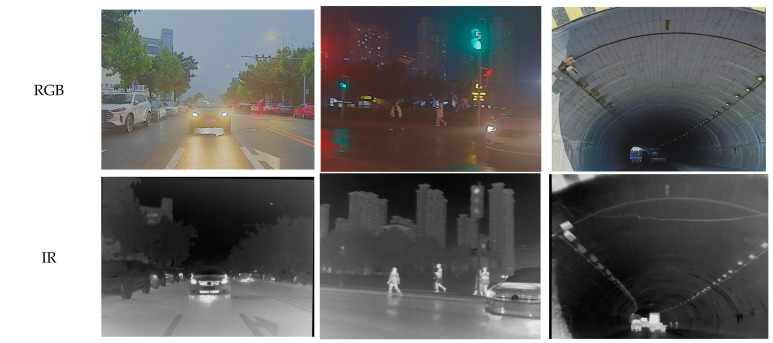

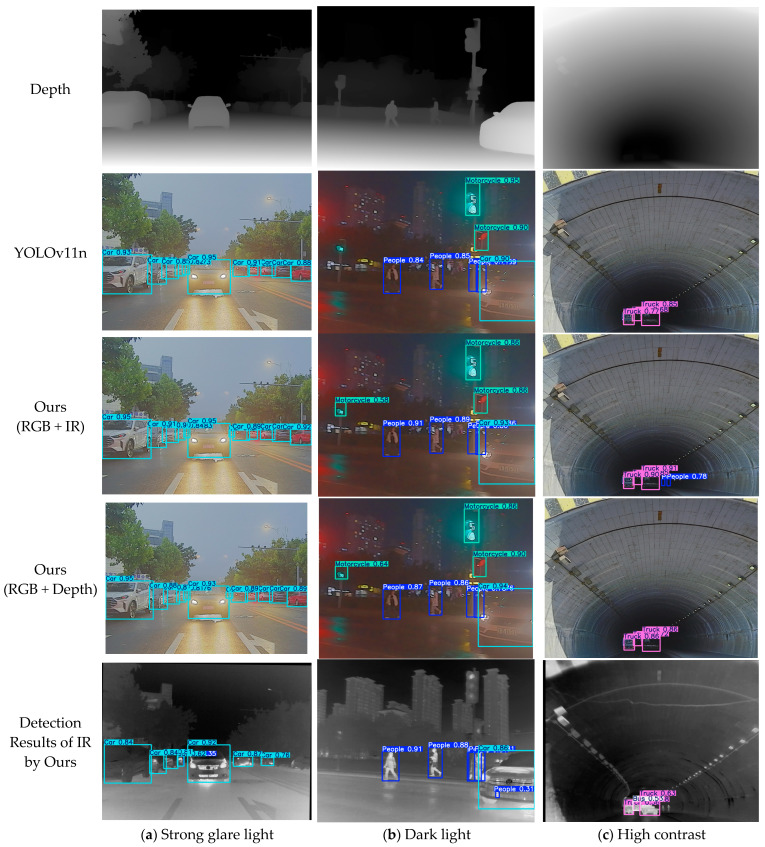
Detection results of the M^3^FD dataset (**a**) Strong glare light; (**b**) dark light; (**c**) High contrast.

The quantitative experiments above have validated the significant advantages of the proposed method in detection accuracy and efficiency. To further investigate the algorithm’s perceptual capabilities in real-world complex scenarios, this section analyzes the model’s performance on typical challenging samples through visual results, elucidating the intrinsic reasons for performance improvements based on the proposed dual-branch fusion mechanism and module design. Detection results of the M^3^FD dataset as shown in Figure 12.

On the M^3^FD infrared-visible dataset, the improved model exhibits stable multimodal fusion advantages under extreme conditions. In scenes with intense glare interference, the enhanced extraction of thermal radiation features from the infrared branch substantially increases detection confidence for roadside vehicles. Under low-light conditions, the fusion of complementary information from visible and infrared modalities enables the detection of traffic signals missed by YOLOv11n. In high-contrast tunnel environments, the infrared branch’s sensitive perception of thermal targets effectively overcomes the limitations of the visible modality, achieving stable localization of pedestrians. These results comprehensively validate the effectiveness and generalization capability of the proposed dual-branch structure and fusion strategy in complex scenarios.

**Figure 13:**
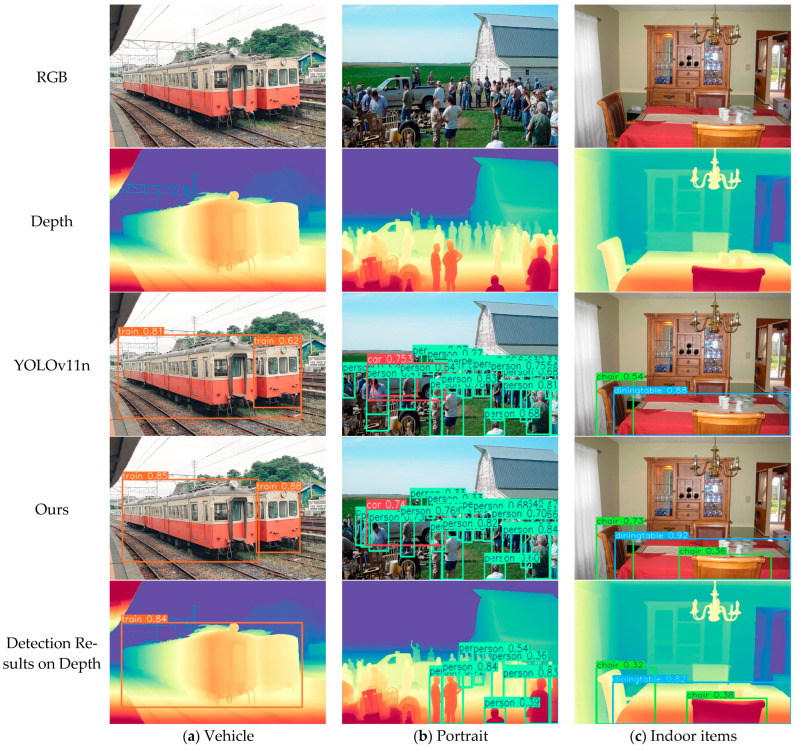
Detection results of the VOC2007 dataset (**a**) Vehicle; (**b**) Portrait; (**c**) Indoor items.

On the VOC2007 dataset, the improved model demonstrates superior geometric perception and detail resolution capabilities. As shown in Figure 13a, when detecting two parallel train carriages, the improved model successfully distinguishes between them leveraging geometric structural information from the depth branch, whereas YOLOv11n erroneously merges them into a single detection due to insufficient spatial perception. In crowded outdoor scenarios (Figure 13b) the feature interaction enhancement mechanism of the DEA module significantly improves target recall under occlusion, enabling complete detection of all pedestrians, while YOLOv11n exhibits noticeable misses. For complex indoor scenes, the improved model not only detects tables and intact chairs but also successfully identifies partially occluded chairs through the multi-level feature fusion of the DEA module, with significantly higher detection confidence than YOLOv11n, demonstrating stronger semantic perception and localization robustness.

## 4. Discussion

The experimental results validate the effectiveness of the proposed dual-branch RGB-D fusion framework in addressing target detection challenges in complex environments. On the VOC2007 and M3FD datasets, the model achieves mAP50 scores of 82.59% and 81.14%, respectively, outperforming the baseline YOLOv11 by 5.06% and 9.15%. These improvements demonstrate the superiority of cross-modal fusion in enhancing detection accuracy. Notably, the model maintains only 2.47M parameters with a computational cost of 19.4 GFLOPs, significantly lower than complex models like RT-DETR, highlighting its efficiency for deployment in resource-constrained scenarios.

Ablation studies provide deeper insights into the contribution of each component. The DBE module’s spatial-channel attention mechanism dynamically weights complementary features from RGB and depth modalities, proving particularly effective in occluded and low-light conditions where single-modal information is insufficient. The DEA module’s dual-encoder strategy optimizes hierarchical cross-modal interactions, enabling the RGB branch to capture texture semantics while the depth branch extracts geometric priors. This complementary fusion significantly enhances robustness in high-interference scenarios such as cluttered backgrounds and illumination variations.

Furthermore, the feasibility of using Depth Anything to generate pseudo-depth maps from monocular RGB images validates the framework’s applicability under data-constrained conditions, expanding its practical utility beyond scenarios requiring dedicated depth sensors. The symmetric dual-branch architecture ensures balanced feature extraction from both modalities, avoiding the dominance of either branch and maximizing the utilization of multi-modal information.

## 5. Conclusions

This study proposes an enhanced YOLOv11-based dual-branch RGB-D fusion framework that effectively addresses accuracy limitations in complex target detection scenarios. By integrating the Dynamic Branch Enhancement (DBE) module for adaptive cross-modal feature weighting and the Dual-Encoder Attention (DEA) module for hierarchical fusion, the framework achieves robust performance in challenging conditions such as occlusion, low illumination, and high interference. The lightweight design, characterized by minimal parameters and computational cost, makes it well-suited for real-time applications in autonomous driving and robotic navigation, where efficient spatial perception is critical.

Future research will focus on three key directions: (1) real-time optimization through model pruning and quantization techniques to meet stringent latency requirements in embedded systems; (2) extension to 3D object localization by incorporating spatial coordinate regression for precise pose estimation; and (3) generalization to diverse sensor configurations, including thermal and LiDAR modalities, to enhance adaptability across varied operational environments. This work establishes a foundation for efficient, high-precision multi-modal object detection in real-world applications.

## Figures and Tables

**Table 1:** The representative studies.

Bibliographic Citation	Description of Method	Advantages
Zhou et al. [6]	Conduct a comprehensive investigation on RGB-D salient object detection and systematically classify traditional and deep learning methods.	Emphasize the complementarity between RGB and deep modalities to provide benchmark datasets and classification frameworks for fusion research.
Peng et al. [7]	The early fusion strategy involves simply concatenating RGB and depth data for fusion at the input or feature layer.	It has low computational overhead, simple processing, and is suitable for resource-constrained scenarios.
Han et al. [8]	The late fusion method independently processes RGB and deep modes and then combines the prediction results.	Retain modal-specific features to enhance robustness against noise.
Li et al. [9]	The ASIF-Net network adopts an interactive fusion scheme and an attention mechanism to dynamically weight cross-modal features.	Enhance feature representation through attention weighting to improve accuracy under low-light and occlusion conditions.
Chen and Li [10]	The PCF network uses complementary perception fusion modules for cross-level interaction.	Explicitly model modal dependencies, reduce fusion ambiguity, and take into account both low-level details and high-level semantics.
Liu et al. [12]	Visual Saliency Transformer (VST) applies the self-attention mechanism to capture long-range dependencies.	Handle the global context of large-scale scenes and enhance performance in complex backgrounds.

**Table 2:** The comparative experimental results on the M^3^FD datasets.

Model	Precision (%)	Recall (%)	mAP50 (%)	mAP50-95 (%)	Params (M)	GFLOPs
YOLOv5n	83.77	64.57	73.12	46.37	2.50	7.1
YOLOv8n	83.63	68.92	74.91	51.40	3.01	8.1
YOLOv10n	76.84	70.15	76.04	47.14	2.27	6.5
YOLOv11n	80.82	65.57	71.89	50.37	2.58	6.3
YOLOv12n	80.79	64.38	70.88	44.42	2.51	5.8
YOLOv13n	73.88	55.61	61.94	38.46	2.45	6.2
RT-DETR	87.24	80.98	81.20	55.00	19.88	57.0
TarDAL (RGB + IR)	-	-	80.70	-	-	14.9
MMDetection-DS (RGB + Depth)	85.01	66.19	75.38	50.20	2.51	19.8
Ours (RGB + IR)	87.03	73.40	81.14	54.69	2.47	19.3
Ours (RGB + Depth)	88.91	68.42	77.37	51.86	2.47	19.3

**Table 3:** The comparative experimental results on the VOC2007 datasets.

Model	Precision (%)	Recall (%)	mAP50 (%)	mAP50-95 (%)	Params (M)	GFLOPs
YOLOv5n	64.89	51.72	56.40	35.66	2.19	5.8
YOLOv8n	78.13	70.83	77.21	57.81	2.69	6.8
YOLOv10n	69.35	56.18	62.46	42.69	2.27	6.5
YOLOv11n	78.85	69.49	77.53	68.35	2.59	6.3
YOLOv12n	80.04	69.25	77.17	58.38	2.51	5.8
YOLOv13n	66.88	53.13	58.55	40.28	2.45	6.2
RT-DETR	81.83	70.57	75.88	58.03	19.90	57.0
Ours	81.22	75.61	82.59	65.27	2.47	19.4

**Table 4:** The comparative experimental results of the accuracy for each category on the M^3^FD datasets.

Class	YOLOv5n	YOLOv8n	YOLOv10n	YOLOv11n	YOLOv12n	YOLOv13n	RT-DETR	Ours (RGB + IR)	Ours (RGB + Depth)
People	66.19	67.74	68.24	66.05	64.61	58.00	75.61	81.60	69.73
Car	87.72	87.92	88.56	87.15	86.46	79.92	87.82	89.89	89.61
Bus	84.63	85.60	84.06	84.24	84.50	75.14	82.75	87.41	88.01
Motorcycle	63.69	65.56	67.94	63.60	59.73	48.37	85.48	75.42	75.42
Lamp	63.47	70.02	74.24	58.38	60.60	50.92	79.74	74.76	69.73
Truck	73.04	72.60	73.18	71.92	69.36	59.27	75.78	77.68	71.77
All	73.12	74.91	76.04	71.89	70.88	61.94	81.20	81.14	77.37

**Table 5:** The comparative experimental results of the accuracy for each category on the VOC2007 datasets.

Class	YOLOv5n	YOLOv8n	YOLOv10n	YOLOv11n	YOLOv12n	YOLOv13n	RT-DETR	Ours
Aeroplane	70.13	84.95	75.27	86.75	84.46	70.13	86.65	88.73
Bicycle	67.38	84.10	75.80	86.13	87.42	70.53	81.63	89.71
Bird	45.27	79.20	56.02	79.69	80.09	52.99	80.49	83.65
Boat	35.04	53.31	38.15	55.29	53.82	34.85	52.43	61.01
Bottle	28.34	57.06	38.71	59.80	53.89	29.03	65.14	75.42
Bus	63.77	82.03	73.30	80.53	81.62	69.05	80.59	86.53
Car	72.85	87.92	76.95	87.01	86.71	73.37	88.48	88.02
Cat	67.37	86.29	72.55	86.30	88.69	65.54	80.19	93.03
Chair	37.59	61.30	42.22	59.97	60.14	34.47	61.17	70.16
Cow	50.02	87.11	53.92	83.91	82.34	49.18	80.25	88.72
Diningtable	59.10	71.04	65.57	72.46	74.78	62.84	74.05	82.73
Dog	50.15	82.01	59.06	78.12	78.82	53.49	75.76	85.70
Horse	76.27	91.85	78.41	92.56	90.24	75.83	82.22	94.03
Motorbike	76.65	89.20	78.06	90.34	90.32	78.49	89.00	92.92
Person	75.31	88.41	78.36	87.62	89.02	76.15	87.46	89.55
Pottedplant	27.27	44.51	32.76	51.70	49.82	26.93	44.45	49.71
Sheep	54.08	78.61	62.51	78.55	81.03	61.70	76.05	84.81
Sofa	47.95	70.75	52.96	69.73	69.73	49.84	69.18	75.16
Train	66.41	86.83	75.05	86.85	84.22	76.99	86.28	87.62
Tvmonitor	57.03	77.67	63.62	77.18	76.34	59.53	74.29	83.40
All	56.40	77.21	62.46	77.53	77.17	58.55	75.88	82.59

**Table 6:** The results of the ablation experiments on the VOC2007 datasets.

Model	Precision (%)	Recall (%)	mAP50 (%)	mAP50-95 (%)	Params (M)	GFLOPs
YOLOv11n	78.85	69.49	77.53	68.35	2.59	6.3
YOLOv11n + A	79.10	69.53	78.03	68.91	2.59	6.3
YOLOv11n + B	80.97	75.03	82.10	64.30	2.47	19.4
YOLOv11n + A + B	81.22	75.61	82.59	65.27	2.47	19.4

**Table 7:** Modality-wise Ablation Analysis on the M^3^FD Benchmark.

Modal			mAP50 (%)	mAP50-95 (%)	Params (M)	GFLOPs
Visible	Depth	Infrared				
√			71.89	50.37	2.58	6.3
	√		67.23	43.60	2.58	6.3
		√	68.70	44.38	2.58	6.3
√	√		77.37	51.86	2.47	19.3
√		√	81.14	54.69	2.47	19.3

## Data Availability

VOC2007: https://opendatalab.org.cn/OpenDataLab/PASCAL_VOC2007 (accessed on 28 April 2007), M^3^FD: https://github.com/JinyuanLiu-CV/TarDAL (accessed on 28 March 2022).
